# Transcriptomic Analysis of Three Differentially Senescing Maize (*Zea mays* L.) Inbred Lines upon Heat Stress

**DOI:** 10.3390/ijms24129782

**Published:** 2023-06-06

**Authors:** Xiaokang Han, Dingyu Zhang, Haibo Hao, Yong Luo, Ziwei Zhu, Benke Kuai

**Affiliations:** State Key Laboratory of Genetic Engineering and Fudan Center for Genetic Diversity and Designing Agriculture, Institute of Plant Biology, School of Life Sciences, Fudan University, Shanghai 200438, China; hanxiaokangpy@163.com (X.H.); zhangdy1225@126.com (D.Z.); hhb199232@163.com (H.H.); ihenauly@163.com (Y.L.); zzw1996@126.com (Z.Z.)

**Keywords:** heat stress, maize, leaf senescence, transcriptome sequencing

## Abstract

Maize, one of the world’s major food crops, is facing the challenge of rising temperature. Leaf senescence is the most significant phenotypic change of maize under heat stress at the seedling stage, but the underlying molecular mechanism is still unknown. Here, we screened for three inbred lines (PH4CV, B73, and SH19B) that showed differentially senescing phenotypes under heat stress. Among them, PH4CV showed no obviously senescing phenotype under heat stress, while SH19B demonstrated a severely senescing phenotype, with B73 being between the two extremes. Subsequently, transcriptome sequencing showed that differentially expressed genes (DEGs) were generally enriched in response to heat stress, reactive oxygen species (ROS), and photosynthesis in the three inbred lines under heat treatment. Notably, ATP synthesis and oxidative phosphorylation pathway genes were only significantly enriched in SH19B. Then, the expression differences of oxidative phosphorylation pathways, antioxidant enzymes, and senescence-related genes in response to heat stress were analyzed in the three inbred lines. In addition, we demonstrated that silencing *ZmbHLH51* by virus-induced gene silencing (VIGS) inhibits the heat-stress-induced senescence of maize leaves. This study helps to further elucidate the molecular mechanisms of heat-stress-induced leaf senescence at the seedling stage of maize.

## 1. Introduction

Heat stress, which is exacerbated by global warming, is considered to be the main cause of global crop losses [1]. To ensure crop production by efficiently improving crop varieties, it is necessary to elucidate the molecular mechanisms of crop response to heat stress [2]. Heat stress affects various physiological processes such as photosynthesis, respiration, and glucose metabolism [3]. Heat stress promotes the accumulation of reactive oxygen species (ROS), which damages the photosynthetic apparatus, particularly PSII, and eventually leads to photoinhibition [4]. In addition, heat stress can damage a plant’s cell membrane system by destroying the protein structure of cell membrane, and malondialdehyde (MDA) produced by membrane lipid peroxidation further aggravates the membrane damage [5]. Several studies have shown that plants produce misfolded proteins under heat stress, which triggers the production of ROS and, ultimately, leads to the senescence or death of plants [6,7,8,9].

Leaf senescence is an important feature of plant responses to abiotic stresses. In recent years, the increasing frequency of extreme heat waves has severely affected plant growth and development. However, the molecular mechanisms underlying leaf senescence induced by heat stress remain largely unrevealed [10]. Studies on wheat have demonstrated that high temperature accelerates leaf senescence, which in turn reduces grain filling time and leads to yield reduction [11]. Maize (*Zea mays* L.) is one of the most planted crops worldwide, with about one-third of the world’s population relying on maize as their main foodstuff. Moreover, maize kernels and stalks are widely used in many other products, including edible oil, starch, petrochemicals, and feed [12].

Maize is affected by heat stress at all stages of growth and development, and studies have shown that maize yields are decreasing each year as the average temperature increases [13,14]. Heat stress can have many adverse effects on maize, such as causing premature leaf senescence, reducing CO_2_ assimilation, and affecting flowering and pollination, and, as a result, affect grain yield [15,16]. High temperatures experienced by maize during the seedling stage can affect its yield due to heat-stress-caused damage. To explore the effects of prolonged heat stress on different maize inbred lines at the seedling stage, we carried out an initial screening and chose three differentially senescing inbred lines (PH4CV, B73, and SH19B), with B73 exhibiting an intermediate senescing phenotype, for transcriptomic analysis. A comparative transcriptome was then conducted to analyze the molecular similarities and differences among PH4CV, B73, and SH19B in response to heat stress, and several key genes were identified. In particular, we detected related genes that might be involved in heat-stress-induced leaf senescence and verified that silencing *ZmbHLH51* could inhibit heat-stress-induced leaf senescence by VIGS. This dataset serves as a foundation for future studies in elucidating the molecular mechanism of heat-stress-induced maize leaf senescence.

## 2. Results

### 2.1. Varied Senescence Phenotypes of Different Maize Inbred Lines in Response to Heat Stress

**Figure 1:**
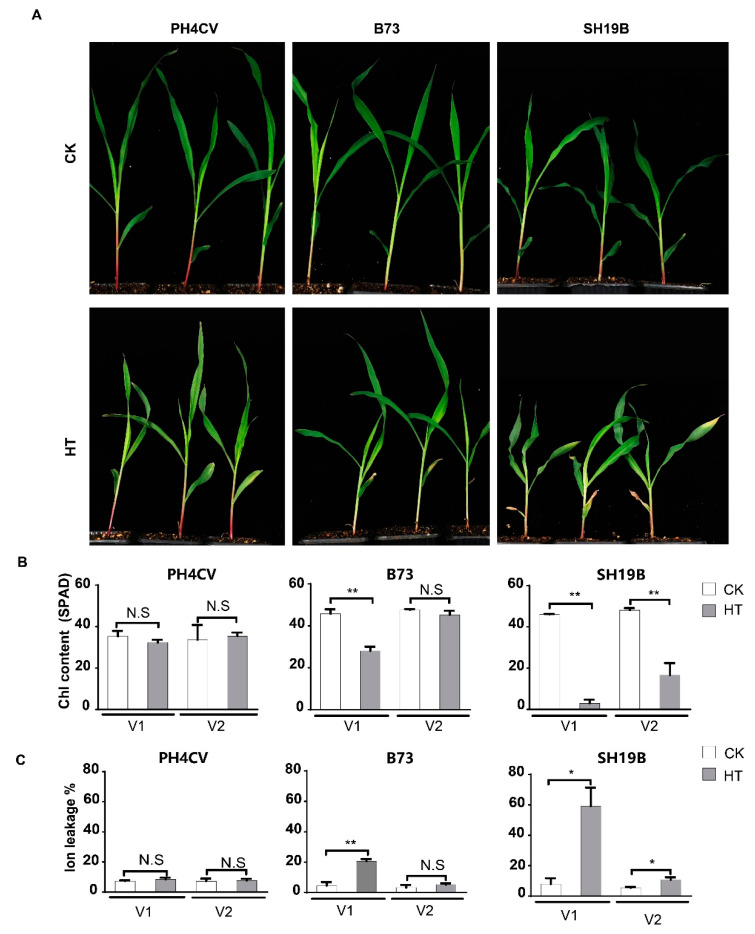
Differentially senesced phenotypes of PH4CV, B73, and SH19B seedlings upon heat treatment. (**A**) Senesced phenotypes of heat treatment (HT) and unstressed (CK) inbred lines; (**B**) chlorophyll contents (SPAD) of the respective organs; (**C**) ion leakage rates (%) of the above organs. V1 and V2 represent the first and second lobes from the bottom, respectively. Error bars represent ±SD (*n* = 3 biological replicates); “N.S” indicates no significant difference, “*” represents *p* < 0.05, “**” represents *p* < 0.01.

Seven maize inbred lines were retrieved from local germplasm collections for the initial analysis of their responsiveness to heat stress. It was demonstrated that the leaves of their seedlings senesced differentially upon high-temperature treatment (42 °C day/35 °C night), and three of them with the distinct phenotypes of senescence—the least-senesced line PH4CV (paternal parent of XY335), the most-senesced line SH19B (CIMMYT inbred line), and the intermediately senesced line B73—were chosen for further analysis (Figure S1). The three-leaf stage seedlings of the three lines were treated with the day/night temperature regime for three days to precisely evaluate their phenotypic response to heat stress. Compared with the untreated controls, SH19B showed a significantly senesced phenotype in both the first lobe (V1) and the second lobe (V2), B73 displayed a significantly senesced phenotype only in V1, while PH4CV exhibited no visible senesced phenotype at all (Figure 1A). Consistently, the chlorophyll contents in both the V1 and V2 of SH19B decreased significantly, whereas a significant decrease in chlorophyll content only occurred in the V1 of B73 and no significant changes in chlorophyll content were detected in the leaves of PH4CV (Figure 1B). Accordingly, the changing trend in the ion leakage rate in the respective organs of both SH19B and B73 was just opposite; as expected, the ion leakage rate in the leaves of PH4CV remained largely similar after heat treatment (HT) (Figure 1C). These observations provoked us to explore the underlying transcriptional changes.

### 2.2. Transcriptome Analysis of the Molecular Basis Underlying the Differentially Senescing Phynotypes in Response to Heat Stress

To explore the molecular basis of differential senescing phenotypes under heat stress, a transcriptome analysis was performed. A total of 12 cDNA libraries were constructed, which consisted of 12 leaf samples of the three inbred lines under HT as well as CK, with two biological replicates for each line. The libraries were then sequenced using Illumina Novaseq 6000 platform. A total of about 85.08 GB of clean data was finally obtained, with an average Q30 value of about 93.7% (Table S1). Using B73 genome (GCF_902167145.1) as a reference, 601 million reads were mapped. There were 84,127,307 (HT_SH19B), 87,847,433 (CK_SH19B), 80,954,154 (HT_B73), 86,897,700 (CK_B73), 87,499,572 (HT_PH4CV), and 90,918,674 (CK_PH4CV) clean paired-end reads used for future analyses (Table S2).

**Figure 2:**
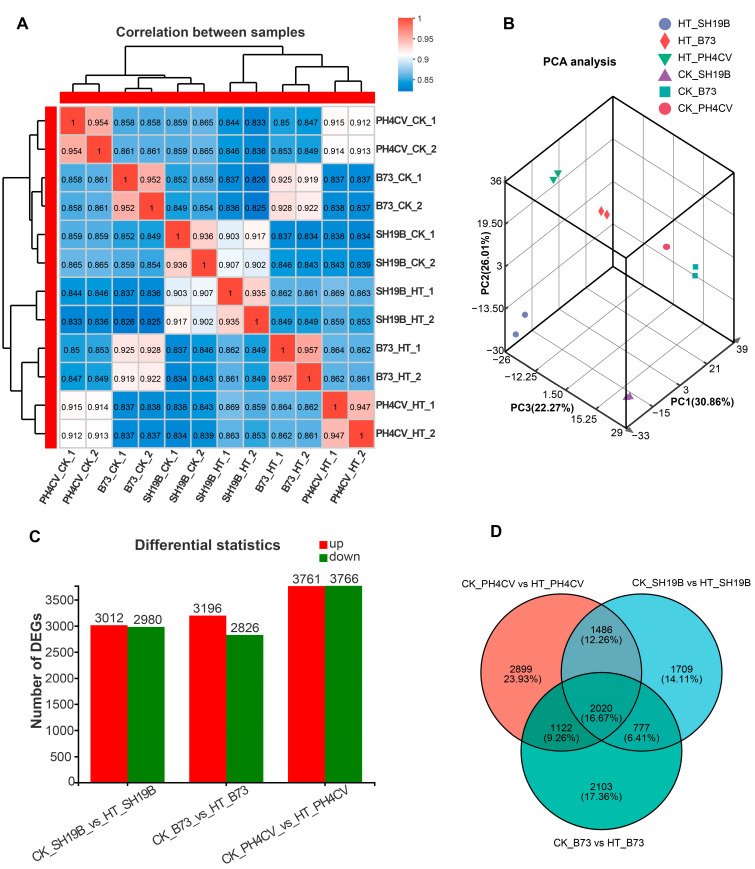
Sample correlation and DEGs analysis. (**A**) Spearman correlation coefficient (SCC) analysis of leaf transcriptomic data of B73, PH4CV, and SH19B under HT and CK conditions; (**B**) principal component analysis (PCA) of sample relationship; (**C**) statistics of DEGs (adjusted *p*-value < 0.05, log2 (fold change) > 1) induced by heat stress; (**D**) Venn diagram of DEGs in three comparisons.

To investigate the global differences in the transcriptomes of PH4CV, B73, and SH19B leaves in response to heat stress, spearman correlation coefficient (SCC) analysis and principal component analysis (PCA) were performed on the average fragments per kilobase million (FPKM) values of all expressed genes in 12 leaf samples. After analysis, the SCC among the biological replicates was around 0.94, indicating a high quality of the replications (Figure 2A). PCA showed that the two biological replicates of each sample were closely clustered, which supported the correlation of the transcriptome sequencing results. In addition, there were differences among the inbred lines and between the HT and CK groups (Figure 2B).

We compared the expressed genes of each inbred line (CK_PH4CV vs. HT_PH4CV, CK_B73 vs. HT_B73 and CK_SH19B vs. HT_SH19B) pairwise to determine the genes that specifically respond to heat stress. The results showed 3012 upregulated and 2980 downregulated genes in SH19B, 3196, and 2826 in B73 and 3761 and 3766 in PH4CV, respectively (Figure 2C, Supplementary File S1). Importantly, it was shown in a Venn diagram that after HT, there were 2020 genes expressed commonly in the three inbred lines, while 2899, 2103, and 1709 genes specifically responded to heat stress in PH4CV, B73, and SH19B, respectively (Figure 2D).

### 2.3. Common Molecular Responses of the Three Inbred Lines to Heat Stress

In order to fully understand the molecular effects of heat stress on the three inbred lines, Gene Ontology (GO) and Kyoto Encyclopedia of Genes and Genomes (KEGG) enrichment analyses were comprehensively performed on the DEGs of CK_PH4CV vs. HT_PH4CV, CK_B73 vs. HT_B73, and CK_SH19B vs. HT_SH19B. It was found that “photosynthesis” (GO:0009768, GO:0009765, map00196, and map00195), “carbon metabolic pathway” (map00500, map00010, map00620, and map00710), “heat stress response” (GO:0009408 and GO:000) 9266), “ROS response” (GO:0000302 and GO:0042542), “osmotic stress response” (GO:0006970), and “salt stress response” (GO:0009651) were commonly enriched in all the three inbred lines (Figures S2 and S3, Supplementary File S2, Supplementary File S3).

**Figure 3:**
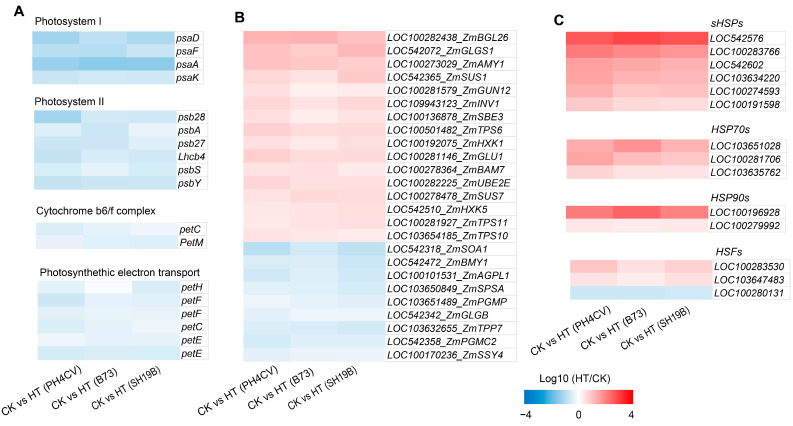
Genes with consistent expression trends in response to heat stress in the three inbred lines. (**A**) Expressions of photosynthesis-related genes (those involved in photosystem I, photosystem II, light complement complex, and photosynthetic electron transport) in the three inbred lines (PH4CV, B73, and SH19B) under heat treatment; (**B**) expressions of sugar-metabolism-related genes; (**C**) expressions of heat-response protein genes (*sHSPs*, *HSP70s*, and *HSP90s*) and *heat-response transcription factor genes* (*HSFs*). Each block represents the ratio of the expression of a gene under heat treatment (HT) to untreated (CK), Log10 (HT/CK). Red indicates upregulation, and blue indicates downregulation. The scale indicates the intensity of the colors.

Other relevant studies revealed that heat stress can cause a decrease in the photosynthesis rate, resulting in a decrease in carbohydrate supply and an increase in carbohydrate demand [17]. We showed the effects of heat stress on photosynthesis-related genes (including those involved in photosystem I, photosystem II, light complement complex, and photosynthetic electron transport) in the three inbred lines through heat maps, and found that photosynthesis-related genes were downregulated in all the three inbred lines (Figure 3A). In addition, we found that the genes related to carbon metabolism were both upregulated and downregulated in the three inbred lines, to varying degrees (Figure 3B), indicating that heat stress had significant effects on photosynthesis and carbon metabolism processes.

Recent studies have found that there is a common response mechanism between heat stress and drought, low temperature, and other stress conditions [5,15,18], indicating a close relationship between heat stress and other types of environmental stress. In addition, ROS is a common response source node under various environmental stress conditions [19]. Studies have shown that ROS can induce the expression of heat-response proteins (HSPs) [20,21]. In this study, we found that HSPs and heat-response transcription factors (HSFs) were significantly enriched in the GO terms of “ heat stress response “ (GO:0009408 and GO:0009266), “ ROS response “ (GO:0000302 and GO:0042542), “osmotic stress response “ (GO:0006970), and “ salt stress response “ (GO:0009651) (Figure S2, Supplementary File S2), and most of them were upregulated in the three inbred lines (Figure 3C). These results demonstrate a common effect of heat stress on the three inbred lines at the transcriptional level and also indicate a general effect of heat stress on maize at the seedling stage.

### 2.4. Distinct Molecular Responses of the Three Inbred Lines to Heat Stress

**Figure 4:**
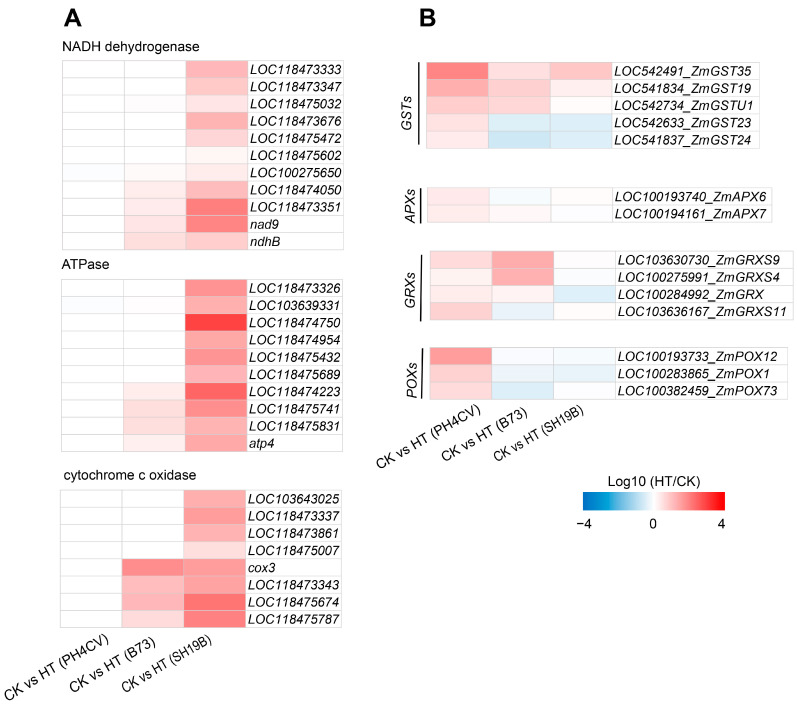
DEGs in response to heat stress in the three inbred lines. (**A**) The differential expression of oxidative phosphorylation pathway genes (including those involved in encoding NADH dehydrogenase, ATPase, and cytochrome c) in the three inbred lines (PH4CV, B73, and SH19B) under heat treatment; (**B**) the differential expression of antioxidant enzyme genes (GSTs, APXs, GRXs and POXs). GST, glutathione S-transferase; APX, ascorbate peroxidase; GRX, glutaredoxin; POX, peroxidase. Each block represents the ratio of the expression of a gene under heat treatment (HT) to untreated (CK), Log10 (HT/CK). Red indicates upregulation, and blue indicates downregulation. The scale indicates the intensity of the colors.

To investigate the differences in the responses of the three inbred lines to heat stress, based on the GO and KEGG analysis results, we found that genes involved in “ATP synthesis” (GO:0009145, GO:0009206, and GO:0009205) and the “oxidative phosphorylation pathway” (map00190) were significantly enriched only in CK_SH19B vs. HT_SH19B (Figures S2 and S3, Supplementary Files S2 and S3). In eukaryotes, oxidative phosphorylation is a biological process in which ATP is produced by combining ADP and inorganic phosphate via the proton gradient produced by the mitochondrial inner membrane. The proton gradient is produced by the catalysis of a variety of enzymes, including: NADH-ubiquinone oxidoreductase, ATP synthase, cytochrome c oxidase, and NADH dehydrogenase [22]. However, oxidative phosphorylation also produces ROS along with ATP, further exacerbating ROS accumulation under heat stress [23]. Several lines of evidence have shown that an increase in the ROS level under heat stress is one of the important factors leading to leaf senescence [24,25,26]. This suggests that heat stress may accelerate the senescence process of SH19B old leaves by producing excessive ROS through the oxidative phosphorylation pathway. Accordingly, we analyzed the differential expression of related genes encoding NADH dehydrogenase, ATP synthase, and cytochrome c oxidase in response to heat stress in the three inbred lines, and found that the expression of these genes was significantly upregulated in SH19B, and some of them were upregulated in B73 as well, while there were no significant changes detected in PH4CV (Figure 4A), indicating that there is a positive correlation between their expression and the senescence degree among the three inbred lines.

Antioxidant enzyme systems can remove ROS and protect plant cells from oxidative damage [14,27]. In this study, a variety of enzymes of an antioxidant defense system were differentially expressed under heat stress, including glutathione S-transferase (GST), ascorbate peroxidase (APX), peroxidase (POX), and glutaredoxin (GRX). We compared the differential expression of these antioxidant oxidase genes among the three inbred lines and found that the five *GSTs* were all upregulated in PH4CV, while *LOC542633_ZmGST23* and *LOC541837_ZmGST24* were downregulated in B73 and SH19B. Two *APXs* (*LOC100193740_ZmAPX6* and *LOC100194161_ZmAPX7*) were upregulated in PH4CV. All four *GRXs* were upregulated in PH4CV, *LOC103630730_ZmGRXS9*, *LOC100275991_ZmGRXS4*, and *LOC100284992_ZmGRX* were upregulated in B73, and *LOC100284992_ZmGRX* was downregulated in SH19B. The three *POXs* were upregulated in PH4CV and downregulated in B73 and SH19B (Figure 4B). These data suggest that the upregulated expression of antioxidant oxidase genes in PH4CV and B73 may be involved in inhibiting ROS-mediated leaf senescence under heat stress.

### 2.5. Responsive Expressions of Senescence-Related Genes in the Three Inbred Lines to Heat Stress

**Figure 5:**
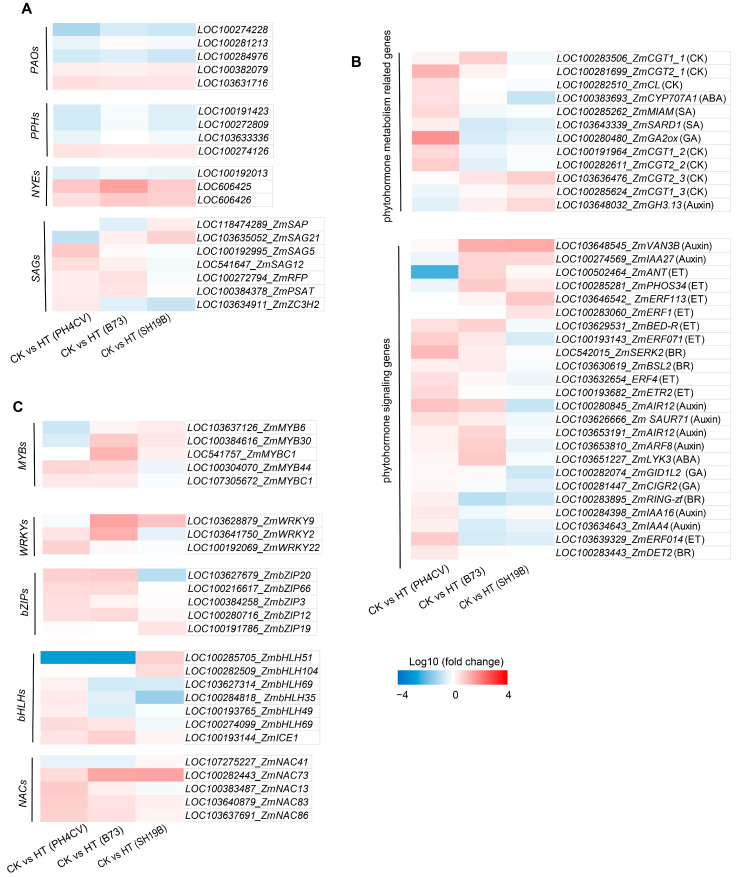
Differential responses of senescence-related genes to heat stress in the three inbred lines. (**A**) Differential expressions of plant phytohormone metabolism and signal transduction genes in the three inbred lines (PH4CV, B73, and SH19B) after heat treatment; (**B**) differential expressions of *TFs* (*MYBs*, *WRKYs*, *bZIPs*, *bHLHs*, and *NACs*); (**C**) differential expressions of *Chl-catabolic genes* (*CCGs*) and *senescence-associated genes* (*SAGs*). Each block represents the ratio of the expression of a gene under heat treatment (HT) to untreated (CK), Log10 (HT/CK). Red indicates upregulation, and blue indicates downregulation. The scale indicates the intensity of the colors.

Leaf senescence-related genes include phytohormone metabolism and signal-transduction-related genes, transcription factors (TFs), *Chl-catabolic genes* (*CCGs*), and *senescence-associated genes* (*SAGs*) [28,29,30]. Phytohormones are closely related to the regulation of leaf senescence. Among them, cytokinins, auxins, and gibberellins (GAs) are involved in the negative regulation of leaf senescence, whereas ethylene (ET), abscisic acid (ABA), salicylic acid (SA), jasmonic acid (JA), and brassinosteroids (BRs) were involved in the positive regulation of leaf senescence [28,29,30]. Figure 5A shows the differential expression of phytohormone metabolism and signaling pathway genes in response to heat stress in the three inbred lines. We detected six cytokinin-O-glucosyltransferases (CGTs) genes and one carboxy-lyase (CL) gene involved in cytokinin metabolism, among which, *LOC100283506_ZmCGT1_1*, *LOC100281699_ZmCGT2_1*, and *LOC100282510_ZmCL* were upregulated in PH4CV or B73 but downregulated in SH19B. *LOC100191964_ZmCGT1_2*, and *LOC100282611_ZmCGT2_2* were upregulated in PH4CV but downregulated in B73 and SH19B. *LOC103636476_ZmCGT2_3*, and *LOC100285624_ZmCGT1_3* were downregulated in PH4CV but upregulated in B73 and SH19B. Abscisic acid 8’-hydroxylase 1 (*LOC100383693_ZmCYP707A1*), gibberellin oxidase 2-beta-dioxygenase (*LOC100280480_ZmGA2ox*), and methyl indole-3-acetate methyltransferase (*LOC100285262_ZmMIAM*) genes were upregulated in PH4CV but downregulated in B73 and SH19B. Moreover, the indole-3-acetic acid-amido synthetase (*LOC103648032_ZmGH3.13*) involved in auxin metabolism was downregulated in PH4CV but upregulated in B73 and SH19B (Figure 5A). Genes related to phytohormone signal transduction showed differential responses to heat stress in the three inbred lines (Figure 5A). Among them, *LOC103648545_ZmVAN3B*, and *LOC100274569_ZmIAA27* involved in auxin signaling and *LOC100502464_ZmANT*, *LOC100285281_ZmPHOS34*, *LOC103646542_ ZmERF113*, and *LOC100283060_ZmERF1* involved in ethylene signaling were upregulated in B73 or SH19B but downregulated in PH4CV; *LOC542015_ZmSERK2* and *LOC103630619_ZmBSL2* involved in BR signaling and *LOC103651227_ZmLYK3* involved in ABA signaling were upregulated in PH4CV or B73 but downregulated in SH19B. In addition, *LOC100281447_ZmCIGR2* involved in GA signaling and *LOC100283895_ZmRING-zf* and *LOC100283443_ZmDET2* involved in BR signaling were upregulated in PH4CV but downregulated in B73 or SH19B. These results indicate that phytohormone metabolism and signaling impose significant effects on leaf senescence induced by heat stress.

TFs such as NACs, bHLHs, bZIPs, WRKYs, and MYBs have been reported to be involved in regulating leaf senescence [31,32,33,34,35]. Here, we showed the differential expression of MYBs, WRKYs, bZIPs, bHLHs, and NACs in response to heat stress in three maize inbred lines (Figure 5B). Among them, *LOC107275227_ZmNAC41*, *LOC100285705_ZmbHLH51*, *LOC100282509_ZmbHLH104*, *LOC100191786_ZmbZIP19*, *LOC103628879_ZmWRKY9*, *LOC103637126_ZmMYB6*, *LOC100384616_ZmMYB30*, and *LOC541757_ZmMYBC1* were upregulated in B73 or SH19B but downregulated in PH4CV. In addition, *LOC100383487_ZmNAC13*, *LOC103627314_ZmbHLH69_1*, *LOC100284818_ZmbHLH35*, *LOC100193765_ZmbHLH49*, *LOC100274099_ZmbHLH69_2*, *LOC103627679_ZmbZIP20*, *LOC103641750_ZmWRKY2*, *LOC100304070_ZmMYB44*, and *LOC107305672_ZmMYBC1* were upregulated in PH4CV or B73 but downregulated in SH19B. These results suggest that these transcription factors, which were differentially expressed in the three inbred lines, may be involved in the regulation of leaf senescence induced by heat stress.

The chlorophyll degradation process has been largely elucidated. It consists of reaction steps catalyzed mainly by three enzymes, NYE/SGR (non-yellowing/stay-green), PPH (pheophytinase), and PAO (pheophorbide a oxygenase) [28,30]. In this study, a total of 3 *NYEs*, 4 *PPHs*, and 5 *PAOs* were detected, and their expression trends in response to heat stress were consistent in the three inbred lines (Figure 5A), indicating that these *CCGs* may not be responsible for the inconsistent senescence phenotypes of the three inbred lines under heat stress. Further analysis showed that *SAGs* expressions were different among the three inbred lines. *LOC118474289_ZmSAP* was upregulated in SH19B but downregulated in PH4CV and B73. *LOC103635052_ZmSAG21* was upregulated in B73 and SH19B but downregulated in PH4CV; *LOC100192995_ZmSAG5*, *LOC541647_ZmSAG12*, *LOC100272794_ZmRFP*, and *LOC100384378_ZmPSAT* were upregulated in PH4CV and B73 but downregulated in SH19B. In addition, *LOC103634911_ZmZC3H2*, which encodes the CCCH zinc finger protein, was upregulated in PH4CV but downregulated in B73 and SH19B (Figure 5C). These are the possible candidate genes involved in heat-stress-induced leaf senescence.

### 2.6. Silencing ZmbHLH51 by VIGS Inhibits Heat-Stress-Induced Leaf Senescence

To temporarily validate the role of the TFs identified in this study on heat-stress-induced leaf senescence, a tobacco rattle virus (TRV)-induced gene silencing (VIGS) system was used. We first used maize PDS (phytoene desaturase) to validate the silencing effect of maize VIGS system [36,37]. It was found that infection with *TRV::ZmPDS* silenced the *ZmPDS*-impaired chloroplast development, resulting in a photo-bleached phenotype (Figure S4), suggesting that the VIGS system used in this study was feasible for silencing maize genes.

**Figure 6:**
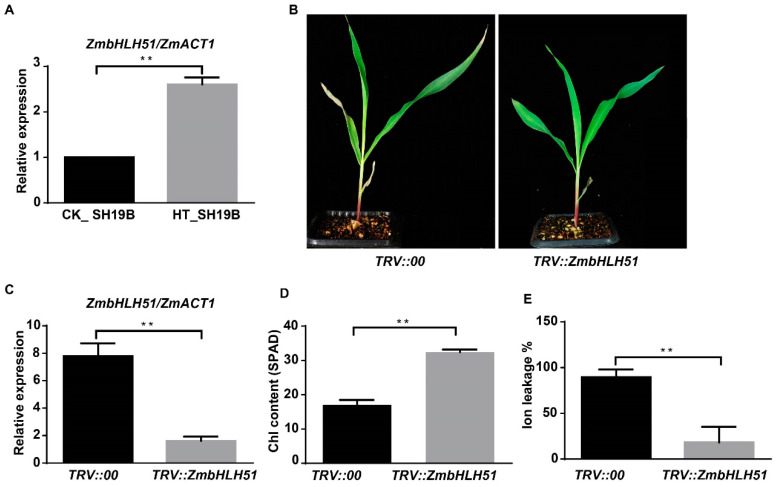
VIGS silencing *ZmbHLH51* inhibited leaf senescence induced by heat stress. (**A**) The relative expression of *ZmbHLH51* in heat treated (HT) and untreated (CK) SH19B, with *ZmACT1* being used as an internal reference; (**B**) maize seedlings infected with *TRV::00* and *TRV::ZmbHLH51* after heat treatment; (**C**) the relative expression of *ZmbHLH51* in *TRV::00* and *TRV::ZmbHLH51* plants after heat treatment, with *ZmACT1* being used as an internal reference; (**D**) chlorophyll content (SPAD) in V2 leaves of *TRV::00* and *TRV::ZmbHLH51* after heat treatment; (**E**) Ion leakage rate (%) in V2 leaves of *TRV::00* and *TRV::ZmbHLH51* after heat treatment. Error bars represent ±SD (*n* = 3 biological replicates); “**” means *p* < 0.01.

In order to determine the inhibitory effect of silencing *TFs* on leaf senescence, four TFs’ genes, *LOC100191786_ZmbZIP19*, *LOC107275227_ZmNAC41*, *LOC100282509_ZmbHLH104*, and *LOC100285705_ZmbHLH51*, which were upregulated in SH19B and downregulated in PH4CV and B73, were initially tested via VIGS experiments. Among the four tested genes, we observed senescence-inhibited phenotypes on the leaves of *TRV::ZmbHLH51* plants in the background of SH19B, consistently within three biological replicates (Figure 6B). Compared with *TRV::00*, the expression of *ZmbHLH51* in *TRV::ZmbHLH51* was greatly reduced (Figure 6C), indicating that *ZmbHLH51* was largely silenced. Leaves of the plants with *ZmbHLH51* silenced contained higher chlorophyll contents and displayed lower ion leakage rates under heat stress (Figure 6D,E). These results indicate that the downregulation of *ZmbHLH51* expression could inhibit heat-stress-induced senescence in maize leaves.

## 3. Discussion

Heat stress can cause leaf wilting or premature senescence of seedling-stage maize plants, decrease a plant’s chlorophyll content, and increase its ion leakage rate [7,18]. In this study, we observed the differentially senescing phenotypes of three maize inbred lines, PH4CV, B73, and SH19B, after heat stress, as evidenced via measurement of the ion leakage rate and chlorophyll content of their leaves. SH19B showed the most obvious leaf senescence symptom, while PH4CV displayed no detectable leaf senescence phenotypes (Figure 1). As far as the DEGs are concerned, there were 2899, 2103, and 1709 independent response genes in PH4CV, B73, and SH19B, respectively, with 2020 co-response genes among them (Figure 2D). Vasseur et al. (2011) pointed out that under heat-stress conditions, the plant metabolic rate increases, resulting in an increase in carbohydrate demand, a decrease in photosynthesis rate, and a decrease in carbohydrate supply [17]. Through GO and KEGG enrichment analysis, we found that the photosynthetic and glucose metabolic pathways in PH4CV, B73, and SH19B were significantly enriched (Figures S2 and S3), suggesting that heat stress could indeed affect both photosynthesis and carbon balance. Other studies have demonstrated that heat stress and drought stress [5,18] or low temperature stress [15] share a common response mechanism. In this study, we found that genes related to heat-stress response, salt-,stress response and osmotic-stress response were significantly enriched in the three inbred lines (Figure S2), indicating a close relationship between heat-stress response and salt-stress and osmotic-stress responses.

Our enrichment analysis further showed that ATP synthesis and oxidative phosphorylation pathways were significantly enriched in SH19B, which provided clues to explore the molecular basis of the differences in response to heat stress among the three inbred lines (Figure S3). Similar to our results, related transcriptome analyses have shown that oxidative phosphorylation plays an important role in response to environmental stress [38,39,40]. Mitochondria produce ATP through oxidative phosphorylation and also produce ROS, which causes damage to plant cells [41]. Studies in Arabidopsis have found that SHOT1 (inhibitor 1 of hot1-4) is a key component of the oxidative phosphorylation complex, and mutant *SHOT1* reduces ROS production, which in turn reduces oxidative damage under heat stress [23]. These studies suggest that the premature senescence of SH19B under heat stress may be caused by excessive accumulation of ROS. ROS is a ubiquitous signal capable of inducing leaf senescence through protein oxidation, enzyme inactivation, altered gene expression, and biofilm breakdown [24,42]. In this study, two GO terms (GO:0000302, GO:0042542) were enriched and related to ROS responses (Figure S2).

The detoxification mechanism of plants is crucial for crop response to heat stress. In this study, a total of three *POXs*, five *GSTs*, four *GRXs*, and two *APXs* were identified, which were involved in heat stress response and were differentially expressed in the three inbred lines (Figure 4B). POXs and APXs are known to break down H_2_O_2_ in cells, thereby protecting them from damage caused by heat stress [43,44]. GSTs can combine glutathione with various heterogeneous compounds to form soluble glutathione S-conjugates to achieve plant detoxification [45]. In addition, GRXs were involved in regulating ROS levels under both normal growth and stress conditions [14]. Recent studies have shown that overexpression of Arabidopsis GRXS17 in maize alleviates heat damage and significantly increases yield compared to non-transgenic maize lines [14]. In this study, most of the antioxidant enzyme genes were upregulated in PH4CV but downregulated in SH19B (Figure 4B), indicating that PH4CV maintained a higher level of antioxidant capacity, better protected the integrity of the cell membrane, and inhibited leaf senescence induced by heat stress.

The increase of ROS level under heat stress is the main cause of *SAGs* expression and leaf senescence [8,24,46]. In this study, we found that multiple *SAGs* responded to heat stress and were differentially expressed in three inbred lines (Figure 5). Several studies have shown that phytohormones play an important role in heat-stress-induced leaf senescence [47,48,49,50]. In this study, we found that the genes related to the metabolism of cytokinins, ABA, GA, SA, and auxin respond to heat stress and are differentially expressed in the three inbred lines (Figure 5A). More importantly, the genes related to ABA, GA, SA, ET, auxin, and BR signaling in response to heat stress were also differentially expressed in the three inbred lines (Figure 5A), indicating that these phytohormone metabolism and signal-transduction-related genes may be involved in heat-stress-induced maize leaf senescence. In addition, TFs are widely involved in complex cascades of signaling induced by hormonal signaling and environmental stresses [51]. In this study, we found that numerous genes of the NACs, bHLHs, bZIPs, WRKYs, and MYBs TF families responded to heat stress and were differentially expressed in the three inbred lines (Figure 5B). Notably, quite a few *SAGs* were differentially expressed in the three inbred lines (Figure 5C), indicating that long-term heat stress may regulate leaf senescence in maize through direct induction of SAGs expression. In Arabidopsis, bHLH family TFs PIF4 and PIF5 were found to effectively promote heat-stress-induced leaf senescence through hormonal signaling [10]. In this study, silencing *ZmbHLH51*, which was upregulated in SH19B in response to heat stress, by VIGS inhibited leaf senescence (Figure 6), suggesting that *ZmbHLH51* is involved in promoting heat-stress-induced leaf senescence. These data provide a foundation for further analysis of the cascade signals of leaf senescence induced by heat stress in maize.

## 4. Materials and Methods

### 4.1. Maize Inbred Lines and Heat Stress

B73, SH19B, and SWL01 inbred lines were kindly gifted by Dr. Yuan Guan (Crop Breeding and Cultivation Research Institute, Shanghai Academy of Agricultural Sciences, Shanghai, China); PH4CV, PHW03, Aijin525 and PHR36 inbred lines were kindly gifted by Dr. Huiyong Li (Cereal Crop Research Institute, Henan Academy of Agricultural Sciences, Zhengzhou, China). The sterilized maize seeds were soaked in water for 1 day and germinated for 14 days under 25 °C, 60% relative humidity, 16 h light/8 h darkness. Two-week-old seedlings were treated under a 42 °C/35 °C day/night cycle for 2 d or 3 d. V1 and V2 leaves experiencing the heat stress for 2 d were collected and subsequently used for transcriptomic analysis. The chlorophyll content and ion leakage rate of maize leaves were measured and calculated by SPAD-502 PLUS and digital conductivity meter (Waterproof ECTestr11+, MultiRange), respectively.

### 4.2. cDNA Library Preparation and Illumina Sequencing

RNA was extracted from 12 leaf samples of three maize inbred lines. RNA quality was monitored by agarose-gel electrophoresis. RNA concentration was checked by Nanodrop2000 (NanoDrop Technologies, Wilmington, CA, USA). RNA integrity was further checked by Agilent 2100 bioanalyzer (Agilent Technologies, Santa Clara, CA, USA). Transcriptome sequencing was performed on Illumina Novaseq 6000 sequencing platform (Majorbio, Shanghai, China).

### 4.3. Transcriptome Sequencing Data Analysis

Fastp was used for quality detection, including deletion of joint sequences, low quality read segments, high N-rate (N indicates uncertain base information) sequences, and excessively short sequences. Then, sequence alignment analysis was performed by TopHat2 (http://tophat.cbcb.umd.edu/, accessed on 15 March 2022) with reference to the maize B73 genome (Zm-B73-REFERENCE-NAM-5.0). Cufflinks software (Version 2.2.1, http://coletrapnelllab.github.io/cufflinks/, accessed on 15 March 2022) was used to assemble and splice the matched sequences and compare them with known transcripts. Genes were annotated by NR (http://ftp.ncbi.nlm.nih.gov/blast/db/, accessed on 20 March 2022), KEGG (http://www.genome.jp/kegg/, accessed on 20 March 2022), Swiss-Prot (http://web.expasy.org/docs/swiss-prot_guideline.html, accessed on 20 March 2022), eggNOG (http://www.ncbi.nlm.nih.gov/COG/, accessed on 20 March 2022), and Pfam (http://pfam.xfam.org/, accessed on 15 March 2022). SCC and PCA were used to analyze the correlation and variation size of samples. RSEM was used to calculate the fragments per kilobase of transcript per million mapped reads (FPKM) [52]. DEGseq2 was used to analyze the identify of DEGs with the criterion of log2 (fold change) > 1 and adjusted *p*-value < 0.05 [53]. Venn was used to analyze the number of overlapping genes between samples. Goatools (https://github.com/tanghaibao/GOatools, accessed on 23 March 2022) and KOBAS (http://kobas.cbi.pku.edu.cn/home.do, accessed on 23 March 2022) were used for GO and KEGG enrichment analysis. By Fisher’s exact test and BH (FDR) multiple test, the corrected *p*-value < 0.05 was considered as a significant enrichment item.

### 4.4. qRT-PCR

The relative expression of maize genes was detected by qRT-PCR. In brief, cDNA synthesis was performed using a RT Kit with gDNA Clean for qPCR II (Accurate Biology, Changsha, China). The transcript levels of each gene were measured using a 20 μL volume of SYBR (Accurate Biology, Changsha, China) on a CFX96 real-time PCR assay system (Bio-Rad, Hercules, CA, USA). qRT-PCR was performed under the following conditions: initial denaturation at 95 °C for 10 min, 95 °C for 20 s, 60 °C for 30 s, and 72 °C for 20 s for 40 cycles. We used 2^−∆∆Ct^ method to calculate the relative expression of RT-qPCR. Primers used for RT-qPCR are summarized in Table S3. *ZmActin* was used as an internal control [54].

### 4.5. VIGS

VIGS experiments were performed as described previously [36,37]. A specific silencing sequence of target gene was screened by the SGN-VIGS tool (https://vigs.solgenomics.net/, accessed on 23 March 2022) and inserted into *pTRV2*. *A. tumefaciens* strain GV3101 carrying *pTRV1*- and *pTRV2*-derived vectors (*TRV2::PDS*, *TRV2::ZmbHLH51*) were cultured in LB medium to (OD600) 1.0. Then, *pTRV1* and *pTRV2* agroinfiltration liquids were mixed in equal volume, adding acetosyringone (20 mg L^−1^), cysteine (400 mg L^−1^), and Tween 20 (5 mL L^−1^). Three-day-old maize seedlings were completely soaked in mixed agroinfiltration liquid, then infiltrated for 10 min with vacuum assistance. The mixture was placed in a flask and co-cultured at 28 °C for 10 h. After co-culture, the seeds were cleaned with sterile water and planted in a light incubator. We verified the silencing effect of the maize VIGS system by ZmPDS. Primers used in VIGS are summarized in Table S4.

### 4.6. Statistical Analysis

In this study, the GraphPad Prism 6.0 software was used to process data and graphs. The SPSS software (version 20; SPSS, Inc., Chicago, IL, USA) was used for analysis of variance. Gene expression heat maps were created in MS Excel using a three-color scale in conditional format.

## Data Availability

The raw datasets generated by transcriptome sequencing in this study are available at BioProject: PRJNA947789 (http://www.ncbi.nlm.nih.gov/bioproject/947789, accessed on 1 April 2023).

## Supplementary Materials

The following supporting information can be downloaded at https://www.mdpi.com/article/10.3390/ijms24129782/s1.
