# Supplementary material for: Transcriptomic Analysis of Three Differentially Senescing Maize (Zea mays L.) Inbred Lines upon Heat Stress
Source: Int J Mol Sci. 2023 Jun 6;24(12):9782. doi: 10.3390/ijms24129782 (PMC10298251; doi:10.3390/ijms24129782)
Supplement: Supplementary file 1 [file ijms-24-09782-s001.zip › Supplementary File.pdf]

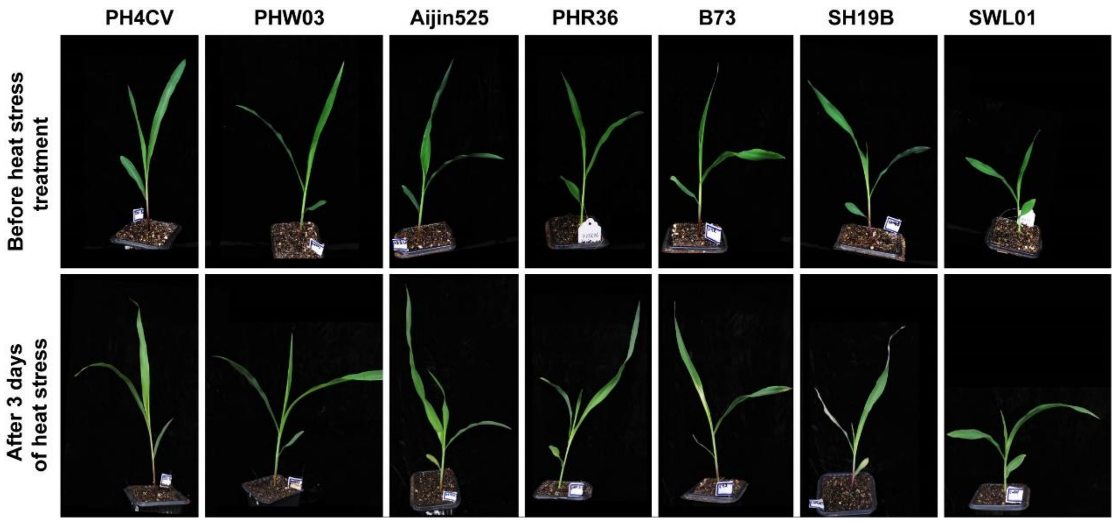

Figure S1 High temperature treatment of different maize inbred seedlings

Table S1 Overview of the sequence assembly after sequencing

Sample	Clean reads	Clean bases	Error rate (%)	Q20 (%)	Q30 (%)	GC content (%)
HT_PH4CV_2	46,840,688	6728360721	0.0255	97.72	93.75	55.36
HT_PH4CV_1	55,332,556	7983560799	0.0254	97.74	93.86	55.73
CK_ PH4CV_2	50,770,178	7421401024	0.0252	97.85	94.08	57.48
CK_ PH4CV_1	52,350,738	7624006548	0.0254	97.76	93.82	56.83
HT_B73_2	45,039,012	6628242089	0.0252	97.81	94.04	55.82
HT_B73_1	41,105,738	6056041940	0.0256	97.68	93.7	55.59
CK_B73_2	46,933,910	6903152502	0.0254	97.78	93.93	56.36
CK_B73_1	45,979,874	6706516175	0.025	97.93	94.31	55.99
HT_SH19B_2	49,722,024	7286026078	0.025	97.91	94.21	55.22
HT_SH19B_1	47,843,076	7010705162	0.0254	97.77	93.9	55.45
CK_SH19B_2	48,882,932	7173025201	0.0253	97.78	93.97	56.64
CK_SH19B_1	52,184,754	7555286216	0.025	97.93	94.32	56.64

Table S2 Summary of reads mapping to the reference genome

Sample	Clean reads	Total mapped	Multiple mapped	Uniquely mapped
HT_PH4CV_2	46840688	40024835(85.45%)	2350878(5.02%)	37673957(80.43%)
HT_PH4CV_1	55332556	47474737(85.8%)	2701570(4.88%)	44773167(80.92%)
CK_PH4CV_2	50770178	44866303(88.37%)	1690258(3.33%)	43176045(85.04%)
CK_PH4CV_1	52350738	46052371(87.97%)	1781891(3.4%)	44270480(84.57%)
HT_B73_2	45039012	42427766(94.2%)	1807935(4.01%)	40619831(90.19%)
HT_B73_1	41105738	38526388(93.73%)	1643912(4.0%)	36882476(89.73%)
CK_B73_2	46933910	43778794(93.28%)	1762350(3.75%)	42016444(89.52%)

CK_B73_1	45979874	43118906(93.78%)	1652792(3.59%)	41466114(90.18%)
HT_SH19B_2	49722024	42997179(86.48%)	2375705(4.78%)	40621474(81.7%)
HT_SH19B_1	47843076	41130128(85.97%)	2253470(4.71%)	38876658(81.26%)
CK_SH19B_2	48882932	42501619(86.95%)	1555796(3.18%)	40945823(83.76%)
CK_SH19B_1	52184754	45345814(86.89%)	1813199(3.47%)	43532615(83.42%)

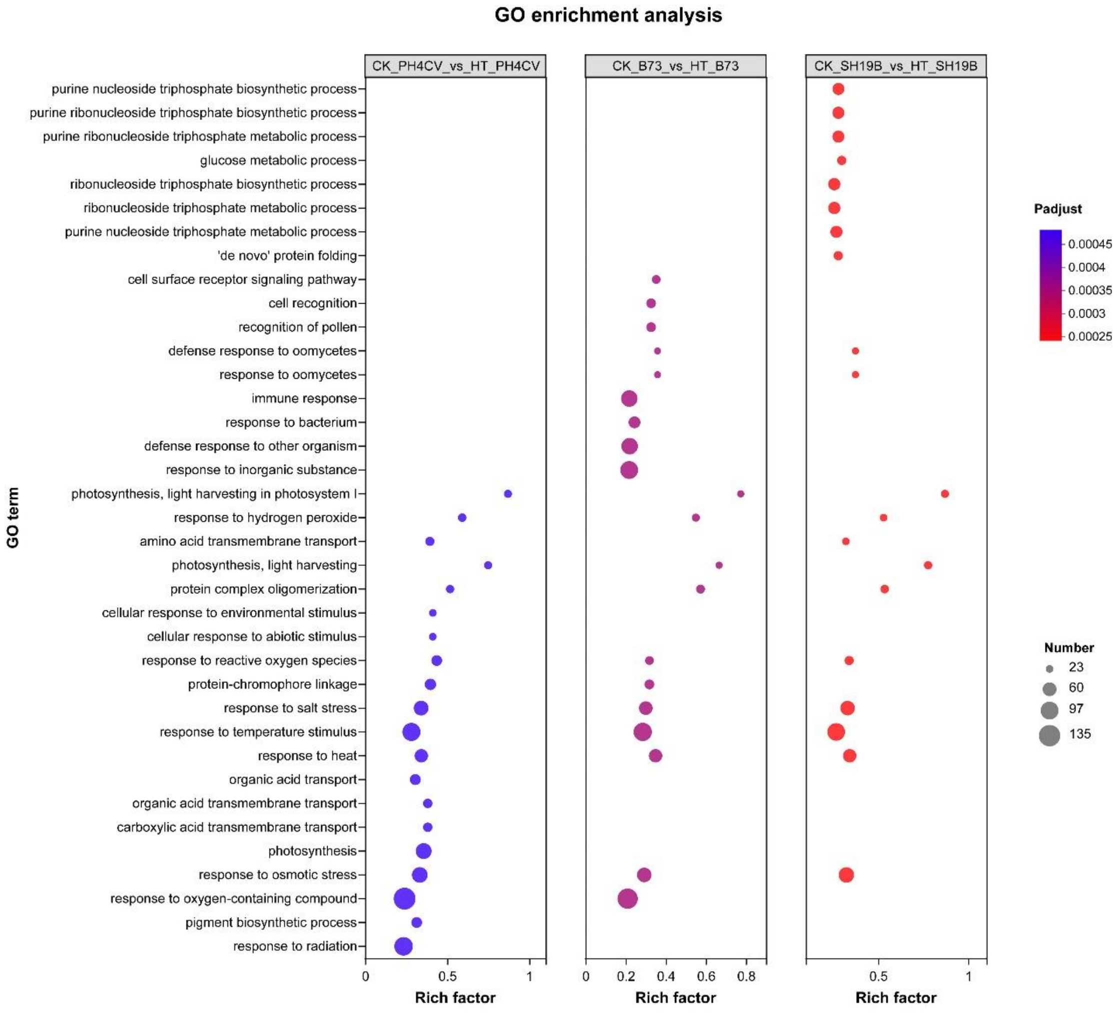

Figure S2. GO enrichment analysis of DEGs in response to heat stress in three maize varieties. The horizontal axis represents the degree of enrichment (rich factor), the vertical axis represents the enriched GO terms; the size of the dot represents the number of differential genes enriched in a GO term; the color of the dot represents the different *P*adjust value; rich factor represents the number of differential genes belonging to a GO term. The higher rich factor is, the higher the enrichment of GO term. CK, untreated control; HT, heat treated.

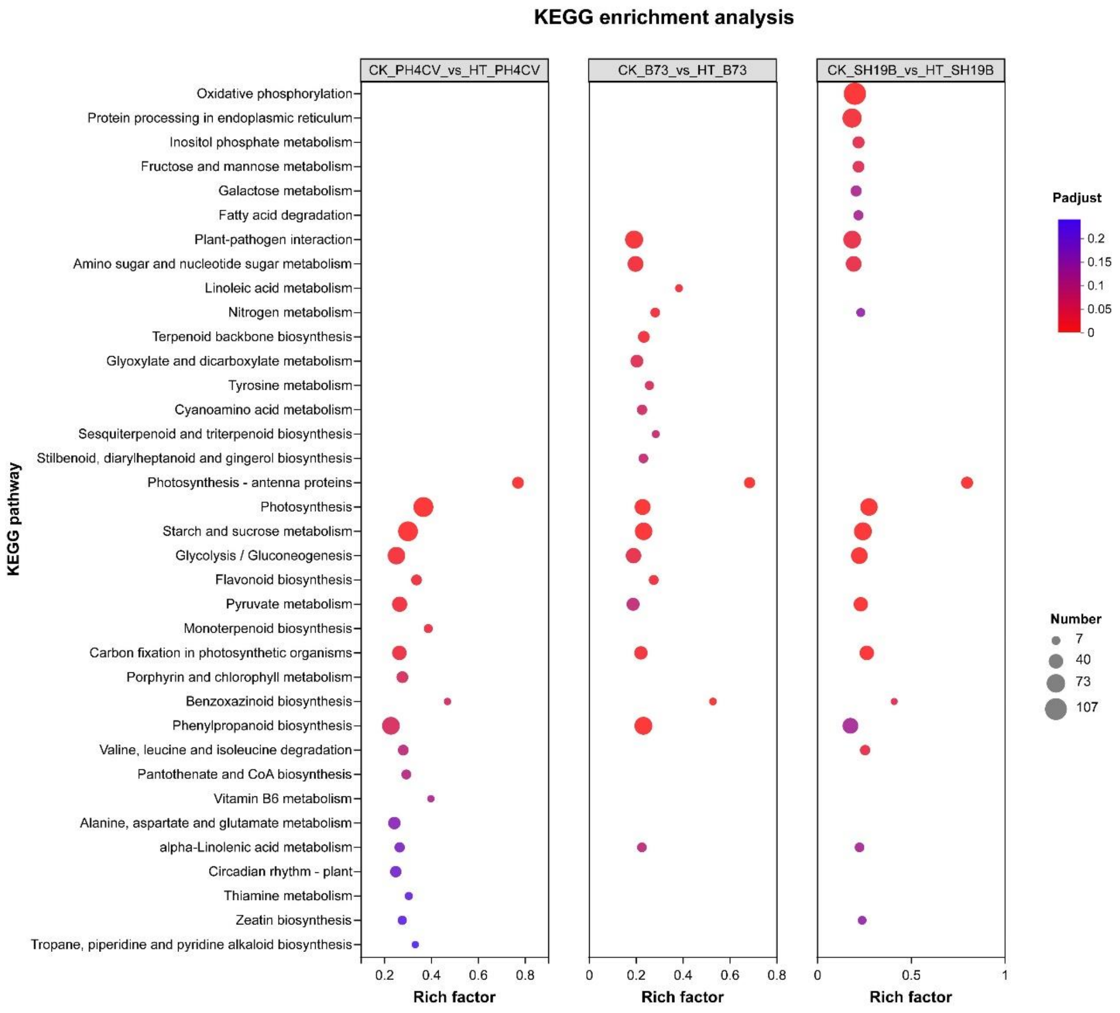

Figure S3. KEGG enrichment analysis of DEGs in response to heat stress in three maize varieties. The horizontal axis indicates the degree of enrichment (Rich factor), and the vertical axis indicates the enriched KEGG pathway; the size of the dot indicates the number of differential genes enriched in a certain KEGG pathway; the color of the dot indicates *P*adjust values; Rich Factor represents the number of differential genes belonging to a certain KEGG pathway. The higher the Rich factor, the higher the enrichment of the KEGG pathway. CK, untreated control; HT, heat treated.

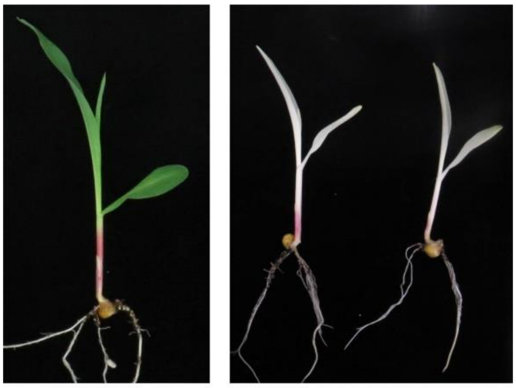

Figure S4. Silence *ZmPDS* through VIGS

Table S3 qRT-PCR primers

Name	Sequence
qZmActin-F	CTGAGGTTCTATTCCAGCCATCC
qZmActin-R	CCACCACTGAGGACAACATTACC
qZmbHLH51-F	AGGATCAATGCTCACCTCGC
qZmbHLH51-R	GCGATCTCGTTGGTCTCTCC

Table S4 Primers for vector construction

Name	Sequence
Zm85705-300-F	AGCCCGGGTGGTTTGCCAAC
Zm85705-300-R	CTGCACAGTACGAAGTCACA
TRV-Zm85705-F	CGACGACAAGACCCTCTGCAGAGCCCGGGTGGTT TGCCAAC
TRV-Zm85705-R	GAGGAGAAGAGCCCTCTGCAG CTGCACAGTACGAAGTCACA
ZmPDS-F	CTAGCCAAGTTATTTCCTGA
ZmPDS-R	GGGACGGGAACTTCTCCTGA
Trv-ZmPDS-F	CGACGACAAGACCCTCTGCAGCTAGCCAAGTTATT TCCTGA
Trv-ZmPDS-R	GAGGAGAAGAGCCCTCTGCAGGGGACGGGAACT TCTCCTGA
